# Serum protein profiles as potential biomarkers for infectious disease status in pigs

**DOI:** 10.1186/1746-6148-8-32

**Published:** 2012-03-22

**Authors:** Miriam GJ Koene, Han A Mulder, Norbert Stockhofe-Zurwieden, Leo Kruijt, Mari A Smits

**Affiliations:** 1 Central Veterinary Institute of Wageningen UR, Lelystad, the Netherlands; 2 Animal Breeding and Genomics Centre, Wageningen UR Livestock Research, Lelystad, the Netherlands

## Abstract

**Background:**

In veterinary medicine and animal husbandry, there is a need for tools allowing the early warning of diseases. Preferably, tests should be available that warn farmers and veterinarians during the incubation periods of disease and before the onset of clinical signs. The objective of this study was to explore the potential of serum protein profiles as an early biomarker for infectious disease status. Serum samples were obtained from an experimental pig model for porcine circovirus-associated disease (PCVAD), consisting of Porcine Circovirus type 2 (PCV2) infection in combination with either Porcine Parvovirus (PPV) or Porcine Reproductive and Respiratory Syndrome virus (PRRSV). Sera were collected before and after onset of clinical signs at day 0, 5 and 19 post infection. Serum protein profiles were evaluated against sera from non-infected control animals.

**Results:**

Protein profiles were generated by SELDI-TOF mass spectrometry in combination with the Proteominer™ technology to enrich for low-abundance proteins. Based on these protein profiles, the experimentally infected pigs could be classified according to their infectious disease status. Before the onset of clinical signs 88% of the infected animals could be classified correctly, after the onset of clinical sigs 93%. The sensitivity of the classification appeared to be high. The protein profiles could distinguish between separate infection models, although specificity was moderate to low. Classification of PCV2/PRRSV infected animals was superior compared to PCV2/PPV infected animals. Limiting the number of proteins in the profiles (ranging from 568 to 10) had only minor effects on the classification performance.

**Conclusions:**

This study shows that serum protein profiles have potential for detection and identification of viral infections in pigs before clinical signs of the disease become visible.

## Background

In present livestock husbandry with increasing requirements for higher health and welfare issues but also tight economical margins, there is a need for tools allowing the early warning for disease. Ideally, easy to perform tools should be available that warn farmers and veterinarians that animals are infected, preferably before the onset of clinical signs. Regular use of such tools may diminish growth retardations and production losses. However, tests for early diagnosis can only be developed when animal-associated "biomarkers" exist that differ between uninfected healthy animals and infected, but not yet diseased, animals. The search for such biomarkers can be performed by two different approaches, either focusing on differences in predefined "candidate" markers, or by comparative fingerprint analysis of "all" components present in a biological sample.

In human medicine extensive research has been performed aiming at the discovery of early biomarkers for different kinds of disease, including cancer. Early diagnosis is important because of increased treatment options and better prognosis when treatments are initiated at an earlier stage [1-3]. In such settings, involving alteration of several pathways and processes, it has been suggested that multiple marker assays lead to an increase in clinical sensitivity and specificity relative to single-marker assays [4]. Also for the early detection of infections in veterinary medicine it has been shown that a combination of protein biomarkers increases the performance, i.e. for transmissible spongiform encephalopathies (TSEs), paratuberculosis, *Dichelobacternodosus *and *Fasciola hepatica *[5-9].

The discovery of potential biomarkers for a number of human and animal diseases has been facilitated by proteomic analysis, some of which have already been commercialized [10]. Comparative proteomic analyses can be performed relatively easily using surface enhanced laser desorption/ionization time-of-flight mass spectrometry (SELDI-TOF-MS) [11]. SELDI-TOF-MS technology includes the use of protein chip arrays that specifically bind intact proteins present in biological samples, such as body fluids or tissue extracts. Arrays may vary in their surface chemistry, for instance they may have hydrophobic or hydrophilic properties, thereby selectively binding proteins. Protein components are solely identified by their specific molecular weights. By comparing SELDI-TOF-MS profiles, protein components that differ in abundance between (groups of) samples can be recognized.

SELDI-TOF-MS generates a profile of peaks representing the relative abundance of each protein component retained on the chip and has a high specificity in distinguishing (groups of) samples. This is especially true when used in combination with a technology to enrich low-abundant proteins, i.e. the Proteominer™ technology, which is based on affinity chromatography using a solid phase combinatorial peptide ligand library. The latter leads to a reduction of the dynamic range of plasma protein concentrations and an improved access to low abundant proteins. The combination of these technologies provides protein profiles representing the relative concentrations of a large number of high- and low-abundant proteins in a biological sample [12,13]. In addition, this technology can be used at a medium throughput scale.

To assess the potential of serum protein profiles as a diagnostic marker for viral infectious diseases in pigs, we used an experimental animal model for porcine circovirus-associated disease (PCVD), an important swine disease mostly known in the manifestation of postweaningmultisystemic wasting syndrome (PMWS). It is, at present, one of the most economically important diseases in swine industry. Although Porcine Circovirus type 2 (PCV2) is regarded as the primary causative agent, PCVD is considered a multifactorial disease. PCV2 pathogenesis appears to be related to the immune-modulatory effects of the virus while other micro-organisms contribute to the clinical signs associated with PCVD. Both porcine parvovirus (PPV) and porcine reproductive and respiratory syndrome virus (PRRSV) have been shown to be associated as co-factors. Experimental co-infections of PPV or PRRSV with PCV2 have fully reproduced PCVAD. These data have been supported by field data in which these viruses have been isolated in association with PMWS [14].

The objective of this study was to explore the potential of serum protein profiles consisting of both high- and low-abundant proteins, as measured by SELDI-TOF-MS, for the diagnosis of early infectious disease status in pigs. To this end the serum protein profiles, obtained from experimentally infected PCV2/PPV, PCV2/PRRSV, and control animals were used to investigate the classification accuracy for different comparisons, i.e. infected versus non-infected, PCV2/PPV versus control, PCV2/PRRSV versus control, and the three-way classification PCV2/PPV, PCV2/PRRSV, and control. In addition, we investigated the classification performance of subsets of protein profiles that varied in the number of used protein components.

## Results

### Clinical signs and pathology

During the course of the experiment, all pigs infected with PCV2 in combination with either PPV or PRRSV developed clinical disease signs with a varying degree of severity. No systemic disease signs were observed in the control group with the exception of a temporary lameness in one pig and paleness in two pigs. Three pigs in the PCV2/PRRSV infected group and one pig from the PCV2/PPV infected group were euthanized for humane reasons at 25 and 26 days post infection. Two pigs died directly after blood sampling, supposedly not related to the experimental infection; one from the control group (at 18 days post infection) and another pig from the PCV2/PRRSV group (at 12 days post infection).

**Figure 1:**
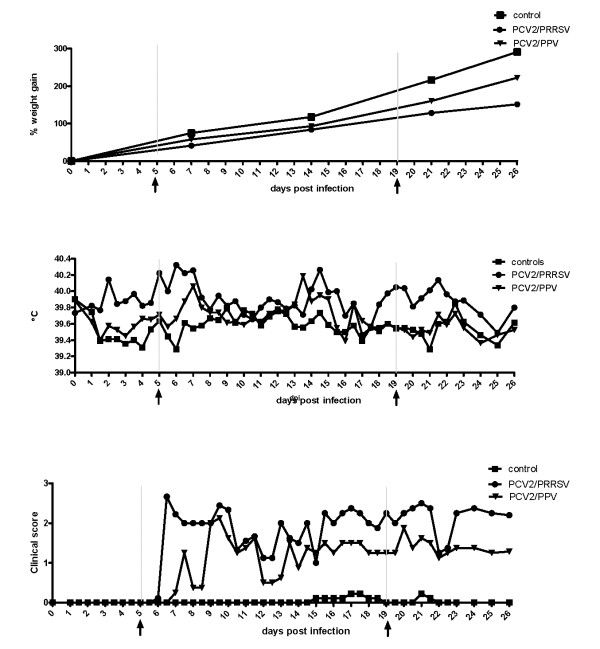
**Body weight gain (A), Body temperatures (B) and clinical scores (C) in experimentally infected groups**. Course of the body weight gain (Figure 1A), body temperature (Figure 1B) and clinical scores (Figure 1C) were recorded in experimental groups of three weeks old, colostrum-deprived piglets inoculated with either a combination of PCV2 and PRRSV (PCV2/PRRSV), PCV2 and PPV (PCV2/PPV), or phosphate buffered saline (controls). For each experimental group consisting of eight (PCV2/PPV) or nine animals (PCV2/PRRSV, controls) data were collected for a period of 26 days post inoculation. Rectal temperatures were measured twice daily and clinical scores were determined based on a collection of predefined clinical symptoms ranging from no disease (0) to severe disease (3). Arrows (↑) indicate time of sampling for SELDI-TOF protein profiling.

Pigs in both the PCV2/PRRSV and the PCV2/PPV infected groups showed signs of wasting with a significant difference in weekly body weight gain compared to the control group. This was consistently seen starting at one week post infection (p < 0.001 and p < 0.05, respectively). Body weight gain was significantly lower in the PCV2/PRRSV infected group compared to the PCV2/PPV infected group in the first week post infection. In the second week post infection, weight gain was similar in both infected groups. Data are shown in Figure 1A.

Mean rectal temperature in pigs in the PCV2/PRRSV infected group increased to febrile temperatures, i.e. rectal temperatures ≥ 40.0°C from two days post infection on for seven days and again elevated mean body temperatures were seen at 14 days post infection and for a period of five days between 18 and 23 days post infection (Figure 1B). In the PCV2/PPV infected group febrile body temperatures were observed incidentally at seven days post infection and at 13 days post infection.

**Figure 2:**
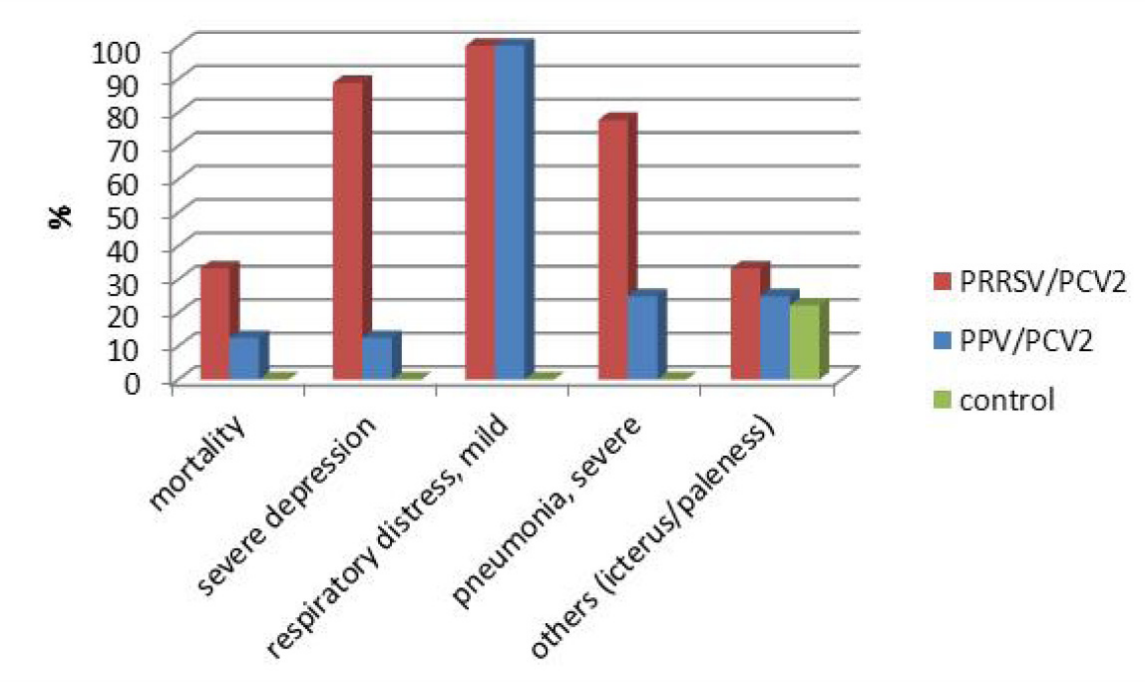
**Clinical symptoms in experimentally infected groups**. The percentage of animals in three weeks old, colostrum-deprived piglets showing diverse signs of disease. Observations were made twice daily during a time frame of 26 days post inoculation with either a combination of PCV2 and PRRSV (PCV2/PRRSV), PCV2 and PPV (PCV2/PPV) or phosphate buffered saline (controls).

Clinical signs started to appear between day six and seven in all pigs from the PCV2/PRRSV infected group and also in a number of pigs from the PCV2/PPV infected group. In the PCV2/PRRSV group the mean clinical score, based on the occurrence and severity of clinical symptoms, reached 2.5 (moderate to severe disease) of a maximum score of 3. In this group severe disease signs started to develop from seven days post infection on (Figure 1C). In the PCV2/PPV infected group the manifestation of disease symptoms occurred slightly later and the mean clinical score was generally lower in this group. Clinical signs as severe depression were observed in more than 80% of all pigs from the PCV2/PRRSV group compared to 10% in the PCV2/PPV group. Whereas respiratory distress was recorded in all infected pigs, 75% from the PRRSV co-infected group showed signs of pneumonia and only about 20% of the PPV co-infected group (Figure 2). A palpable increase of the size of the inguinal lymph nodes was found in all PCV2/PRRSV infected pigs from 12 days post infection on and in three pigs from the PCV2/PPV infected group from 12, 19 or 21 days post infection on.

At necropsy, typically for PMWS, inguinal lymph nodes and also other lymph nodes were enlarged in all infected pigs and the mean weights of the inguinal lymph nodes in both infected groups were higher than in the control group (PCV2/PRRSV vs. control, p < 0.03; PCV2/PPV versus control, p < 0.08). In the PCV2/PRRSV infected group two of nine pigs had a macroscopically identifiable pneumonia, although a moderate to severe interstitial pneumonia was found in eight of nine pigs based on histology. In the PCV2/PPV infected group macroscopic changes were restricted to increased size of the inguinal lymph node and kidney, liver or lung changes in a few pigs. Histologically, most striking was a slight to moderate hepatitis in seven of eight pigs. In five of 17 PCV2 infected pigs lymph node depletion was observed, in others a hyperplasia was more prominent.

After termination of the experiment, tissue samples were tested for the presence of PRRSV and PCV2 nucleic acid detection by PCR. All infected animals showed strong positive results for PCV2 in lymph nodes, lung and spleen. Although in control animals no PCV2 nucleic acid was detected in pharyngeal swabs throughout the study, low levels of PCV2 nucleic acid were found in single or several tissue specimens of six control animals. PRRSV nucleic acid was detected in lungs of all PCV2/PRRSV infected animals but not of the other groups. No PCR testing has been performed for PPV.

### Acute phase proteins

**Figure 3:**
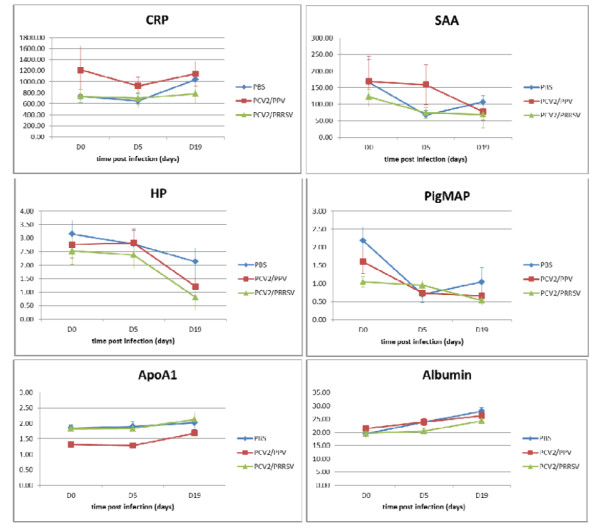
**Acute phase protein levels in experimentally infected groups**. Mean levels of C-reactive protein (CRP), serum amyloid A(SAA), haptoglobin (Hp), pig major protein (PigMAP), α-Lipoprotein (ApoA1), and albumin were determined in three weeks old, colostrum-deprived piglets inoculated with either a combination of PCV2 and PRRSV (PCV2/PRRSV), PCV2 and PPV (PCV2/PPV), or phosphate buffered saline (controls).

Results of acute phase proteins levels are summarized in Figure 3. At day five p.i. levels of acute phase proteins did not differ significantly compared to levels in sera collected prior to inoculation, except for pig major protein (PigMAP, p = 0.004) and albumin (p = 0.01) in the control group and PigMAP in the PCV2/PPV infected group (p = 0.035). More significant differences were observed at day 19 p.i. compared to levels at day zero for three acute phase proteins; haptoglobin (Hp) in the PCV2/PRRSV group (p < 0.001), PigMAP in PCV2/PPV (p = 0.023) and PCV2/PRRSV infected animals (p = 0.021), and albumin in PCV2/PPV (p = 0.002), PCV2/PRRSV infected animals (p = 0.003), as well as in the PBS treated control group (p = < 0.001).

Some statistically significant differences were observed comparing different experimental groups. At day zero, PigMAP levels in animals from the PCV2/PRRSV group (p = 0.013) and α-Lipoprotein (ApoA1) levels in the PCV2/PPV group (p < 0.001) were significantly different compared to the control group. At day five p.i., only ApoA1 (control group versus PCV2/PPV group, p = 0.004), and at day 19 only albumin (control group versus PCV2/PRRSV group, p = 0.032) were discriminative.

### SELDI-TOF proteomics

Serum protein profiles were obtained on day zero as well as on day five post infection, before clinical symptoms became apparent. A third set of protein profiles was obtained at day 19 post infection, when all animals from both experimental infections showed clear signs of disease. Using the Protein chip Data Manager software a total of 586 protein peaks were identified and subjected to further statistical analysis. It should be noted that these 586 protein peaks may represent a lower number of proteins, as a certain overlap may be present among the results obtained with the three types of arrays that have been used.

Protein profiles of serum obtained from all animals prior to infection (day 0) were tested for differences. No statistical significant differences could be observed between animal groups, indicating that in this respect, the three groups of animals were very similar at the time of infection.

### Overview of comparisons

We performed multiple analyses, comparing the three experimental groups: animals inoculated with (i) PCV2 and PPV (PCV2/PPV); (ii) PCV2 and PRRSV (PCV2/PRRSV); and (iii) PBS (controls), at three time points. An overview of these analyses is given in Table 1. The comparisons can be categorized as follows: (1) infected animals (PCV2/PPV together with PCV2/PRRSV) versus non-infected control animals, (2) PCV2/PPV infected animals versus control animals or PCV2/PRRSV infected animals versus control animals, (3) PCV2/PPV, PCV2/PRRSV, and control animals as three distinct groups to explore the discriminatory power of serum protein profiles to distinguish the different infection models.

**Table 1:** Overview of the statistical analyses (A-H) with the accompanying data sets that were used

	Analysis			**Day 5 p.i**.			**Day 19 p.i**.	
2/3way	comparison	Day p.i.	Control	PCV2/ PPV	PCV2/ PRRSV	Control	PCV2/ PPV	PCV2/ PRRSV

2-way	Infected (A2) versus non-infected (A1)	5, 19	A1^a^	A2	A2	A1	A2	A2

	PCV2/PPV versus control	5	B1	B2				

	PCV2/PRRSV versus control	5	C1		C2			

	PCV2/PPV versus control	19				D1	D2	

	PCV2/PRRSV versus control	19				E1		E2

	PCV2/PPV versus control	5, 19	F1	F2		F1	F2	

	PCV2/PRRSV versus control	5, 19	G1		G2	G1		G2

3-way	PCV2/PPV, PCV2/PRRSV, control	5, 19	H1	H2	H3	H1	H2	H3

^a ^Serum protein profiles of animals in groups with the same code have been clustered for statistical analysis.

Different comparisons (A-H) were carried out, including serum protein profiles of animals from different infection groups and/or time points after infection. SELDI-TOF-MS profiles were obtained from three week old piglets that were experimentally infected with either a combination of PCV2 and PRRSV (PCV2/PRRSV), PCV2 and PPV (PCV2/PPV), or phosphate buffered saline (controls). Statistical analyses were based on data from day five post infection (p.i.), day 19 p.i., or data from both days combined as indicated in the table.

For each comparison the number of significantly different protein peaks in their profiles was determined as well as the classification accuracy. Additionally we tested whether reducing the number of protein peaks in a profile affected the accuracy of classification. Next to whole protein profiles consisting of 586 protein peaks, we also tested profiles with 500, 200, 100, 50, 20, and 10 protein components. For this, proteins were ranked by significance and those with highest significance were selected.

### Number of significant different protein peaks

For each comparison the number of significantly different protein peaks is shown in Table 2. Based on a maximum p-value of 0.01, the number of differentially expressed protein components ranged from 15 (PCV2/PPV versus control) to 59 (PCV2/PRRSV versus control). For a number of comparisons, molecular masses of the most significant differently expressed protein components are summarized in Table 3.

**Table 2:** Overview of the number of significant differentially expressed protein components in different analyses

		Number of significant proteins	
**Analysis^a^**	**Day p.i**.	**p < 0.01**	**FDR < 0.05**

Healthy versus diseased	5, 19	49	13

PCV2/PPV versus control	5	26	1

PCV2/PRRSV versus control	5	36	1

PCV2/PPV versus control	19	15	-

PCV2/PRRSV versus control	19	17	-

PCV2/PPV versus control	5, 19	19	-

PCV2/PRRSV versus control	5, 19	59	43

PCV2/PPV, PCV2/PRRSV, control	5, 19	45	12

a: See Table 1 for description of analysis.

SELDI-TOF-MS protein profiles were generated from three week old piglets experimentally infected with either a combination of PCV2 and PRRSV (PCV2/PRRSV), PCV2 and PPV (PCV2/PPV), or phosphate buffered saline (controls). The number of significantly differentially expressed protein components with p < 0.01 or with False Discovery Rate (FDR) < 0.05 for different analyses are indicated.

**Table 3:** Most significantly differentially expressed protein components as characterized by mass:charge (m/z) value

Analysis							
**Infected vs controls**		**PCV2/PPV vs** **control**		**PCV2/PRRSV vs control**		**PCV2/PPV**, **PCV2/PRRSV, and** **control**	

**m/z**	**q-value**	**m/z**	**q-value**	**m/z**	**q-value**	**m/z**	**q-value**

5540	0.025	54287	0.055	5540	0.010	5540	0.034

8720	0.032	10602	0.158	8720	0.013	147239	0.034

54287	0.032	8565	0.209	147239	0.014	8720	0.034

8565z	0.032	80028	0.283	17175	0.019	8720	0.034

19966	0.034	8720	0.283	34135	0.019	34135	0.034

14540	0.034	187711	0.283	19966	0.019	54287	0.037

10436	0.037	2357	0.283	17171	0.019	64263	0.037

11021	0.037	10516	0.283	10436	0.019	187711	0.037

10726	0.037	10805	0.283	11021	0.019	5542	0.037

2357	0.042	27621	0.283	5542	0.019	8193	0.037

The ten most significantly differently expressed protein components for four analyses as characterized by mass:charge (m/z) value with their q-value (in Daltons) are summarized. SELDI-TOF-MS Protein profiles were generated from three week old piglets experimentally infected with either a combination of PCV2 and PRRSV (PCV2/PRRSV), PCV2 and PPV (PCV2/PPV), or phosphate buffered saline (controls). The figures are based on SELDI-TOF-MS data from day five and 19 post infection combined. The overlap in protein components within different comparative analyses is due to the use of different SELDI-TOF-MS protein chip arrays.

As expected, based on the False Discovery Rate (FDR), which may be more appropriate than p-values as it accounts for multiple testing, the amount of differentially expressed protein components was reduced. In a number of analyses, none of the protein components showed a significantly different expression based on an FDR < 0.05. In general, more protein components were differentially expressed comparing PCV2/PRRSV infected animals versus non-infected control animals as opposed to PCV2/PPV infected versus control animals as shown in Table 2. Combining data of day five and day 19 post infection (p.i.) increased the number of significantly differentially expressed protein components.

### Two-group classification

#### Infected animals versus non-infected animals

In this analysis, PCV2/PPV along with PCV2/PRRSV infected animals were marked as infected, whereas control animals at day five and day 19 are regarded as non-infected (Table 1). Classification results are summarized in Table 4.

**Table 4:** The number of animals correctly classified as either infected or non-infected

	**Day 5 p.i**.		**Day 19 p.i**.	
**number of proteins** **components**	**non-infected**	**infected**	**non-infected**	**infected**

10	4 (9)	10 (16)	1 (7)	14 (15)

20	6	12	3	14

50	7	14	3	14

100	7	12	4	15

200	6	13	4	14

500	6	14	3	14

586	6	14	4	14

The number of correctly classified animals (infected versus non-infected) at day five and day 19 post infection using increasing numbers of SELDI-TOF-MS protein components. Serum protein profiles were generated from three week old piglets experimentally infected with either a combination of PCV2 and PRRSV (PCV2/PRRSV), PCV2 and PPV (PCV2/PPV), or phosphate buffered saline (controls). In the top row, for each experimental group the total number of animals included in the analysis is specified between brackets.

At day 19 post infection, both PCV2/PPV and PCV2/PRRSV infected animals displayed evident signs of illness with 14 of 15 (Sensitivity (Se) = 93.3%; specificity (Sp) = 57.1%) infected animals having significantly different serum protein profiles compared to non-infected animals. Moreover at day five, before any disease symptoms were apparent, 14 of 16 (Se = 87.5%; Sp = 66.7%) infected animals could be recognized based on their serum protein profiles.

Interestingly, preselecting protein peaks slightly increased the classification accuracy; using the 50 (day five post infection) or 100 (day 19 post infection) most significant differently expressed proteins resulted in the highest number of correctly classified animals. When the number of proteins was further limited to ten, a decrease in correctly classified animals was observed at day five (Se = 62.5%;Sp = 44.4%). However at day 19 post infection, profiles based on the ten most significant proteins could still identify 93% (= Se) of the infected animals, although only one of the seven non-infected animals was correctly classified (Sp = 14.3%) (Table 4).

#### Two-group classification: PCV2/PPV versus control or PCV2/PRRSV versus control

Serum protein profiles of PCV2/PPV infected animals were compared with profiles of non-infected control animals on day five and day 19 using either data from a single time point or combining data from both days. Similarly, profiles of PCV2/PRRSV infected animals were compared with control animals. Results are summarized in Table 5.

**Table 5:** Number of animals correctly classified according to infection status, evaluating two distinct classes

Separate/ combined	number of used protein markers^1^	Day 5 **p.i**.		**Day 19 p.i**.		Day 5 **p.i**.		Day 19 **p.i**.	
		
		PCV2/ PPV	Control	PCV2/ PPV	Control	PCV2/ PRRSV	Control	PCV2/ PRRSV	Control
Separate	10	2 (7)	5 (9)	4 (8)	5 (7)	8 (9)	6 (9)	5 (7)	6 (7)

	20	2	3	5	5	8	6	5	6

	50	2	5	6	5	6	6	5	6

	100	2	5	6	3	7	7	5	6

	200	1	5	6	3	8	8	4	6

	500	1	5	6	2	8	7	5	5

	586	1	5	6	2	8	7	5	5

Combined	10	2 (7)	5 (9)	7 (8)	3 (7)	7 (9)	8 (9)	5 (7)	6 (7)

	20	2	7	6	4	8	8	6	5

	50	2	8	5	6	7	9	5	6

	100	2	6	6	5	7	9	6	6

	200	2	6	8	4	7	9	6	5

	500	3	6	7	4	7	8	5	6

	586	3	6	7	5	7	9	5	6

The number of correctly classified animals according to infection status (2-way classification), based on serum protein profiles at day five and day 19 post infection (p.i.). The classification results are presented using either data of each day separately or combined. Protein components were selected based on their differential expression in piglets infected with PCV2/PPV, PCV2/PRRSV and control piglets, using those with the lowest p-values. In the top row, for each experimental group the total number of animals included in the analysis is specified between brackets.

With regard to the classification of infected versus non-infected animals at day five post infection, results were poor for the PCV2/PPV group as only one of seven (Se = 14.3%; Sp = 55.6%) infected animals could be distinguished from control animals by profiles consisting of 586 protein components. The classification performance of serum protein profiles was much better for PCV2/PRRSV infected animals (Se = 88%; Sp = 77.8%) at day five post infection.

As expected, overall results were better at day 19 post infection. Six of eight (Se = 75%; Sp = 28.6%) PCV2/PPV and five of seven (Se = 71.4%; Sp = 71.4%) PCV2/PRRSV infected animals could be distinguished from non-infected control animals by serum protein profiles using 586 protein components. This reflects the clinical signs, which were very similar for animals in both groups at 19 days post infection.

Combining the data of both days slightly increased the classification accuracy as compared to profiles from either day five or day 19 post infection. This is especially true for the control groups as shown in Table 5 (Sp ranges from 66.7% - 100%).

Again, using a preselection of the most significantly different protein peaks generally led to comparable or even better classification accuracy, similarly as described for the classification of infected versus non-infected animals.

### Three-group classification

To assess the power of serum protein profiles as a diagnostic marker for specific infections, we explored the classification performance of serum protein profiles to distinguish between the three distinct animal groups (i) non-infected control animals, (ii) PCV2/PRRSV and (iii) PCV2/PPV infected animals) in one analysis, i.e. three-group classification. As expected, three-group classification as shown in Table 6 gave similar results compared to the two-group classification, but with lower classification accuracy.

**Table 6:** Number of animals correctly classified according to infection status, evaluating three distinct classes

		Day 5			Day 19	
**number of protein** **components**	**PCV2/PPV**	**Control**	**PCV2/PRRSV**	**PCV2/PPV**	**Control**	**PCV2/PRRSV**

10	2 (7)	5 (9)	3 (9)	4 (8)	3 (7)	1 (7)

20	4	6	5	6	3	3

50	2	7	8	5	4	3

100	1	7	6	5	4	3

200	1	7	4	5	4	2

500	2	6	6	5	3	2

586	2	5	6	5	4	2

The number of correctly classified animals according to disease status (3 way classification), based on serum protein profiles consisting of variable number of protein components. Serum protein profiles were obtained at day five and 19 post infection (p.i.) and data of both time points were combined for the statistical analysis. Protein components were selected based on their differential expression in piglets infected with PCV2/PPV, PCV2/PRRSV and control piglets, using those with the lowest p-values. In the top row, for each experimental group the total number of animals included in the analysis is specified between brackets.

Table 7 shows the contingency tables for day five and 19 for true disease status and classification based on serum protein profiling using 50 most significant protein peaks. At day five, almost all (8/9) PCV2/PRRSV infected animals were classified correctly. A single PCV2/PRRSV infected animal was misclassified as PCV2/PPV infected. On the other hand, only two of seven PCV2/PPV animals were identified correctly. One was improperly classified as PCV2/PRRSV infected, while four animals could not be discriminated from non-infected animals. However at 19 days post infection, five of eight PCV2/PPV infected animals were accurately classified, while only three of seven PCV2/PRRSV infected animals could be identified based on their serum protein profiles. The other four animals were misclassified as PCV2/PPV infected. Control animals, when misclassified, were labeled as PCV2/PPV infected animals, but never as PCV2/PRRSV infected.

**Table 7:** Contingency table showing the classification results according to infection status, evaluating three distinct classes

		Day 5 p.i.^1^			Day 19 p.i.^1^		
		**True status**			**True status**		

		**PCV2/** **PPV**	**Control**	**PCV2/** **PRRSV**	**PCV2/** **PPV**	**Control**	**PCV2/** **PRRSV**

Classification results based on SELDITOF MS data	PCV2/PPV	2	2	1	5	3	4
	
	Control	4	7	0	1	4	0
	
	PCV2/PRRSV	1	0	8	2	0	3

^1 ^The p-value of the one-sided Fisher exact test was < 0.001 and 0.095 at day 5 and 19 respectively.

Contingency table for 3-way classification, showing the number of correctly and incorrectly classified animals at day five and 19 post infection (p.i.), respectively. Classification was based on SELDI-TOF-MS serum protein profiles from piglets at day five and 19 after inoculation with either PCV2/PPV, PCV2/PRRSV or phosphate buffered saline (controls). Data of both time points were combined for the statistical analysis using 50 preselected protein components. Protein components were selected on their differential expression, using those with the lowest p-values.

In conclusion, PCV2/PRRSV infected animals could well be distinguished from control animals as early as day five, while PCV2/PPV infections were best distinguished from non-infected control animals at day 19. Classification results showed a very high significance at day five (P < 0.001) and were near significance at day 19 (P = 0.095). It can be concluded that based on SELDI-TOF protein profiles, at day five post infection PCV2/PRRSV infected animals are easier to distinguish compared to PCV2/PPV infected animals.

## Discussion

Livestock health is an important issue for farmers and veterinarians as well as for consumers. It has an important economic drive as it affects productivity. In addition, from the animal welfare perspective there is a need for parameters that can objectively measure abnormalities or deterioration of health, preferably in an early stage of disease. Although the number of animals involved in this study was rather small, this study shows that SELDI-TOF MS profiles of high- and low-abundant serum proteins have potential as diagnostic markers for early detection of viral infections in pigs. We also show that classification of animals using ridge penalized partial least squares analysis of the protein profiles might be a powerful approach for this.

We realize that in the current setting, we collected data under standardized experimental conditions. However, to make a useful and robust multi-marker test based on protein profiles, test development and validation should include the use of animals that originate from different breeds, different farms, different time points post infection and from animals with different disease history. Since such factors will create additional variation in protein profiles, larger sample sizes will be required. In this respect it is promising that combining profiles of day five and day 19 resulted in improved classification accuracies, suggesting that protein fingerprints of different time points after infection show similarities, which might be utilized under field conditions when time of infection varies.

### Correlation between SELDI-TOF MS results and clinical data

The aim of this study was to explore the potential of quantitative data of high- and low-abundant serum protein components as measured by SELDI-TOF-MS for early detection and diagnosis of viral infectious diseases in pigs. The experimental infection model (true status) of the animal groups was considered as the golden standard, which was reflected by representative clinical signs. The infection status was confirmed by PCR, after termination of the experiment.

The SELDI-TOF MS serum protein fingerprints reflect the (pre-)clinical status of the two different disease courses having a comparable disease outcome. Among PCV2/PRRSV infected animals, body temperatures rose after day five post infection, while clinical scores increased sharply from day six onwards when infected animals were depressed and showed clear respiratory distress symptoms. Among PCV2/PPV infected animals body temperatures started to rise from day six onwards, while clinical scores did not increase until day seven post infection, typically consisting only of mild depression. It can be speculated that for PCV2/PPV infected animals protein fingerprints taken at day five may have been too early for diagnostic purposes, in contrast to the PRRSV/PCV2 infected animals.

It should be noted that among the control animals no apparent clinical signs were observed. The low content of PCV2 viral DNA in tissue samples of some control animals are considered to have been "false positive" test results. However it cannot be fully excluded that, in spite of containment measures during the animal experiment, some level of cross contamination with the PCV2 virus occurred in the control group.

### Using a whole-protein profile approach instead of candidate proteins

In the discovery of biomarkers a targeted approach is often used, aiming at a selection of predefined biomarkers based on current knowledge of the biological function of proteins or known associations. An example for such approach is the use of acute phase proteins for early diagnostic markers for infections. As levels of these proteins change early in the process of infection or tissue trauma, they have been suggested as suitable biomarkers [15,16].

In the present study, the analysis of acute phase proteins led to disappointing results (Figure 3). Similar to our experience, experiments conducted by Heegaard et al. found large between-animal variation and major differences in prechallenge concentrations between experimental groups [16], limiting the use of acute phase proteins as general disease markers.

As an alternative, we explored a whole protein profile approach using SELDI-TOF-MS and comparative fingerprint analysis of whole protein profiles present in blood samples and studied its value for early disease diagnosis. This approach was chosen because it enables the identification and selection of "reactive profiles" without any prior knowledge of the biological functions of the components constituting the profiles. Although knowledge of biological function of proteins could have additional value and can be used as biological validation, it is not mandatory. Another advantage is that protein profile fingerprints enable the monitoring of quantitative changes rather than determining particular threshold levels of individual serum proteins.

A major challenge in the discovery of protein biomarkers from blood is the vast difference in concentration between high- and low-abundant proteins. With traditional analysis methods, the high-abundance proteins usually dominate the proteome profiles, making the identification of less abundant protein components more challenging. Different strategies have been developed to eliminate some of the most abundant proteins from blood serum or plasma [17]. Here we applied the Proteominer™ fractionation kit from Bio-Rad. It is based on a bead-bound random peptide library that provides a vast amount of different binding sites for different proteins. Since there is only a small number of ligands that can bind to the same protein, this limits the number of identical high-abundant protein components that can bind to the bead-bound library. The combination of depletion, enrichment and fractionation through the Proteominer™ fractionation kit used in this study has led to the detection of a high number of differentially expressed high- and/or low-abundance proteins (as shown in Table 2) underlining the value of this technique. Focussing on low-abundance proteins, rather than the classical plasma proteins, might be a more promising approach since low abundant biomarkers may include proteins that either leak into the plasma from different tissues as a result of the infection or that play a role as signal molecules.

### Classification of infected versus non-infected animals

The difficulty with multiple disease classification is that large sample sizes are required. Therefore, classifying animals as infected and not-infected or diseased and not-diseased is probably a first starting point. In this study, no disease symptoms were yet apparent at day five, except that a number of pigs in the PCV2/PRRSV group had elevated body temperatures. Notably, 14 of 16 (87.5%) infected animals could be recognized based on serum protein profiles. As expected, at day 19 post infection results were even better (93.3%). Further analysis will have to provide information whether these differences are due to inflammatory processes or other viral -host interactions. From experimental studies increases in IFNγ secreting cells and interleukin 10 have been shown as early as 7 and 10 days post infection, respectively [18-20].

Also classification according to distinct infection models showed promising results. However, the lower sensitivity as revealed by Table 6 suggests that classification with respect of specific infectious diseases will be challenging. Interestingly, the classification performance on both sample days for infected animals was superior compared to non-infected animals. This probably reflects the normal variation in serum protein profiles among "healthy" animals which is relatively large compared to infected animals in this small cohort. Also Batxelli-Molina et al. found more extensive variation in serum protein profiles from non-infected animals compared to infected animals [6].

It has been shown that in clinical settings multiple marker assays have increased sensitivity and specificity compared to single-marker assays [10]. It may be speculated that increasing the number of markers leads to a further improvement of the diagnostic performance. Indeed, the number of protein components that were statistically significant in differential expression between the distinct animal groups (as shown in Table 2) correlated well with the classification accuracy: the higher the number of significant protein markers between the groups, the better the classification accuracy. However, our findings also suggest that extending protein profiles to more than about 20 markers does not substantially increase classification accuracy. In our case, limiting the number of (preselecting) proteins from whole protein profiles of 586 to the ten most significant differentially expressed components did only marginally decrease the classification accuracy. Also the small number of animals used in this study limits the power of a high number of proteins in their contribution to classification accuracy. This indicates that although there might be quite a number of proteins markers associated with the disease status, performance of profiles seems to be more affected by the predictive value of individual proteins than by the number of proteins included in the profile.

### Statistical methods

For evaluation of SELDI-TOF data a decision tree method is frequently used. However, to analyse complete sets of multiple protein peaks, more sophisticated statistical methods are required. We used ridge penalized partial least regression to classify animals, which is superior to decision tree analysis when there are many proteins contributing to the classification or, in other words, when many proteins are likely to be different between infected and non-infected animals. Partial least squares techniques have also been applied in disease classification in humans [2,21]. The significance testing of individual proteins was equivalent to the approach used by Batxelli-Molina et al. [6] and Barr et al. [5] for testing the significance of proteins in the diagnosis of prion diseases.

In this case, we used one-leave-out cross-validation, because of the very limited number of animals per class. Due to the fact that one animal is left out, the unbalance in animals per disease class may be larger. Nevertheless, two-group as well as three-group classifications were quite successful, although with lower accuracy in the latter. Clearly larger sample sizes are necessary to improve the classification accuracy to more than 90% sensitivity and specificity required for diagnostic purposes.

### Towards development of biomarkers for livestock health

This study shows the potential of protein profiles in combination with advanced statistical methods to distinguish infected from non-infected animals, providing etiological information as well. Such an approach may be valuable in the diagnosis of infectious diseases in the early stage of disease. In this study we examined sera from animals experimentally infected with PCV2 in combination with either PPV or PRRSV. As shown in Table 3 a number of protein components were significantly differentially expressed in multiple comparisons. For instance protein with mass:charge 8720 was evident in all four analyses. Such proteins may be regarded as key candidate markers and further investigation is warranted.

In follow-up studies it would be of interest to explore the classification of animals according to aetiology, such as bacterial, viral, and parasitical infections. Also differentiation according to disease stage, i.e. acute versus chronic or affected organ system could be useful. The ultimate goal might be the development of assays for health versus disease as opposed to specific etiologic agents.

The advances of proteomic technologies and promising study results have fed the hope to obtain biomarkers for improved and faster diagnostics. Due to the high costs and required technical skills, spectrometry has traditionally been limited to research settings. However, it is now increasingly used for diagnostic purposes in routine settings for the identification of infectious microorganisms [15]. SELDI-TOF-MS is a promising tool to determine protein profiles at medium throughput level and at reasonable costs. In human medicine, proteomic methods are increasingly used for early diagnosis of diseases [20]. In addition, it appears that the challenges of multiplexing such tests (e.g., on arrays) are sufficiently daunting that quantitative mass spectrometry may have value as an additional format for multiplexing protein measurements in the future given aggressive technology development. A major disadvantage for mass diagnostics as required in livestock veterinary medicine is the invasive procedure of blood sampling to get appropriate test material for analysis of biomarkers in serum. An alternative for the use of sera would be to explore the potential of protein profiles in easy to access biological samples like saliva, urine or faeces. Also, the recent developments in the field of micro- and nanotechnology have seen a rapid surge in interest in electronic devices for medical implants for in vivo health monitoring. In the human biomedical field several promising prototypes are emerging, for example for monitoring of patients with chronic cardiac or neurological diseases [22]. Similar developments may be expected for the veterinary health care sector.

## Conclusions

In this study the potential of quantitative protein profiles by SELDI-TOF MS for early diagnosis of viral infections in pigs was explored. Results from serum of pigs experimentally infected with a combination of PCV/PPV and PCV2/PRRSV indicate that SELDI-TOF protein profiles have potential for detection of (viral) infection in pigs in early phase of the disease. The accuracy of classification of infected versus non-infected animals was good, as 88% of the infected animals could be classified based on the serum protein profiles at day five post infection, that is before clinical symptoms became apparent. At day 19 post infection, 93% of the infected animals were classified as such. Results for PCV2/PRRSV were superior compared to PCV2/PPV infected animals, especially at day five post infection. The lower specificity, both at day five (67%) and day 19 post infection (57%), probably reflects the variation in serum protein profiles among non-infected animals. Limiting the number of proteins in the profile generally had minor effects on the classification accuracy. Accuracy of three-way classification was less than that of two-way classification. It can be concluded that SELDI-TOF MS protein profiles may have potential as biomarker for early diagnosis of viral infections in animal husbandry.

## Methods

### Experimental infection

The animal experiment was according to Dutch law approved of by the Animal Ethical Committee of CVI (trial code 2008056c). Animals: Twenty- six, colostrum-deprived piglets from a conventional breeding line (TOPIGS 20™) of three weeks of age were housed in three different animal rooms with HEPA filtered supply and exhaust air filtration. Piglets were tested negative by PCR assay for PCV2, PPV and PRRSV prior to start of the study and allocated to three groups, which were either inoculated with PBS, or a combination with either PCV2/PRRSV or PCV2/PPV. Virus inocula: Tissue-culture propagated PCV2b strain 1324 (2^nd ^passage), isolated in 2002 in the Republic of Ireland from a pooled tissue homogenate from a PMWS diseased animal and PPV strain 1005 (8^th ^passage) were kindly supplied by Prof. G. Allan, University of Belfast. The 7^th ^passage of PRRSV strain Ter-Huurne, a EU-strain of PRRSV, propagated on lung macrophages was used. The titer of the PCV2 virus inoculum was 2 × 10^5 ^TCID_50_/ml, of the PPV virus inoculum 2 × 10^6 ^TCID_50_/ml and of the PRRSV virus inoculum 1 × 10^6 ^TCID_50_/ml. On day zero, individual pigs received either the PBS sham inoculum, or the virus pools in a volume of three ml each. For this, aerosols of the inocula were produced by a commercial, gravity-fed, single trigger airbrush (Evolution™, Harder&Steenbeek, NL) with a nozzle of 0.2 mm, creating an aerosol with 90% of droplets smaller than 99 μm in diameter, 50% of droplets smaller than 50 μm, and 10% droplets smaller than 26 μm. The aerosol was administered intranasally alternately to each nostril during inspiration phases. Pigs were weighed weekly and followed clinically for a period of 27 days after infection. Rectal temperatures were measured twice daily. Inguinal lymph nodes were palpated daily to monitor increase in size and clinical symptoms were recorded by using pre-defined identifiers. These identifiers or a combination of identifiers were used to define a clinical score per day as no disease (0), mild (1), moderate (2) or severe (3) disease. Serum blood samples were taken from the external jugular vein at days 0, 2, 5, 7, 9, 12, 15, 19 and 22. After coagulation, serum was separated by centrifugation at 2000 g for 10 min. and stored at -80°C until analysis. At day 27, pigs were euthanized and a full necropsy was performed. Tissue specimens were taken for virus nucleic acid detection by polymerase chain reaction (PCR) analysis as described [14]. Briefly, RNA and DNA were extracted from organ suspensions using the QIAmp blood and tissue kit (Qiagen, Westburg, the Netherlands) for DNA and the High Pure RNA isolation kit for RNA (Roche diagnostics, Germany) according to the manufacturer's recommendations. To quantify the amount of PCV2 DNA copies in organ samples, a real-time fluorescent-probe PCR with the light-Cycler probes (LC red 640 - ATC TCA TCA TGT CCA CCG CCC AGG A) (FL fluorescein -CGT TGT ACT GTG GTA CGC TTG ACA GT) and the primers (1391; 5'-CTC CCC TGT CAC CCT GGG TG -3' and 1577; 5'-CTC TCC CGC ACC TTC GGA TAT-3') amplifying a 186-bp fragment from the cap gene of PCV2 were used. The viral RNA concentration of PRRSV was assessed by a reverse transcription real time PCR with the following primers: 5'-GAT GAC RTC CGG CAY C -3' (forward); 5'- CAG TTC CTG CGC CTT GAT -3' (reverse) exerted on a MX3005 (Stratagene) machine.

### Acute phase proteins

The serum concentration of haptoglobin was measured by use of an assay based on haemoglobin-haptoglobin binding [23] while serum CRP and pig MAP [24] and SAA concentrations were assayed by ELISA [16]. The concentration of Apo A1 was determined by radial immunodiffusion [16] and albumin was measured using a dye-binding assay for this protein on an automated biochemistry analyser (Prestige Analyser, Trio-Diagnostics Ltd, York). Assays for porcine APP were performed by ReactivLab Ltd, (Glasgow, UK).

### Serum enrichment and fractionation

To detect the proteins present in low levels it is advisable to remove the most abundant proteins first [13]. Recently, a new method for enriching low-abundance proteins has been commercially available. This technology is known under de trade name of ProteoMiner^® ^(BioRad, Veenendaal) and is based on the use of a combinatorial peptide binding library, which affinity-captures and amplifies the low abundance proteome [12]. ProteoMiner^® ^treatment was performed according to manufacturer's recommendation. Briefly, 525 mg bulk beads swelled by rehydration with 10 ml 20% (v/v) aqueous EtOH. 100 μl of this beads solution is after washing with water and PBS in a 96-well filter plate (Pall-5039) mixed with 200 μl centrifuged serum for two hours at 4°C. After binding and washing the beads three times with 200 μl PBS, the proteins were eluted three times with 20 μl of each of the four elution reagents; fraction1 (1 M NaCl, 20 mM HEPES pH7.5), fraction2 (200 mM glycine pH2.4), fraction3 (60% ethylene glycol), fraction4 (33% isopropyl alcohol, 16.7% acetonitrile, 0.1% TFA). Between every elution the beads where mixed for 5 minutes at room temperature, and centrifuged 1 min at 1000 g to collect the eluent from filter plate to a collection plate.

### Protein profiling

Protein examination was performed according to the manufacturer's instructions. Briefly, a volume of 100 μl of the 10-fold diluted fractions, in appropriate binding buffer depending of the array, were incubated on the spots of three type of ProteinChip arrays (BioRad). A cation exchange (CM10) array with CM10 binding buffer (100 mM sodium-acetate pH 4.0), a copper-coated IMAC array with IMAC binding buffer (0.1 M sodium phosphate, 0.5 M NaCl pH7), and a reverse phase (H50) array with H50 binding buffer (10% acetonitrile, 0.1% trifluoroacetic acid (TFA)).

After 60 minutes incubation the arrays were washed three times with 200 μl appropriate binding buffer, followed by a wash with 200 μlMilliQ water.

After the arrays were dry, 2 × 1 μl of a saturated solution of sinapinic acid (SPA) in 50% acetonitrile (v/v), 0.5% trifluoroacetic acid (v/v) was added. The mass spectra of the proteins captured on the chips were recorded with the PCS4000 ProteinChip array reader (BioRad) by using ProteinChip Data Manager software 3.5.0.

Previous to the measurements, the mass spectrometer was checked using the OQ kit (BioRad) for high voltage conditioning, detector calibration, detector sensitivity, mass drift, mass resolution, and mass accuracy. For calibrating, the All-in-One Protein Standard II (Biorad) was used.

The resulting protein profiles, obtained from the time-of-flight mass spectrometry spectra were analysed for differences in expression using Bio-Rad ProteinChip Data Manager, version 3.5.0 with the integrated Biomarker Wizard™ cluster analyses software (Biorad). First, peaks with a signal to noise ratio higher than five were selected. These were clustered with peaks having similar masses in other profiles with signal to noise ratios higher than two. Before cluster analyses, the baseline was subtracted and profiles were normalized using total ion current.

### Data processing

After the identification of the peaks and normalization of the profiles, a total of 586 proteins from 26 animals were subjected to statistical analysis. For a number of proteins data were unavailable at some time points. Two animals died during the experiment and only data on day zero and day five were available. Additionally some missing values existed for subsets of proteins (e.g. CM10 or IMAC). Animals with absent data on a specific day were excluded only for that day.

#### Significance testing proteins

Initially an ANOVA was performed for each protein, to detect significant differences between disease groups (PCV2/PPV, PCV2/PRRSV, versus control, or infected versus uninfected animals) using an F-test. Multiple testing increases the risk of false positives. To reduce the risk of false positives, the false discovery rate (FDR) was used. The FDR was set to 5% and the obtained P-values of the F-test were converted into so-called q-values using the package 'qvalue' in R [25]. The different comparisons are listed in Table 1. In addition, we tested also for differences prior to infection at day zero. No proteins showed significant differences between animal groups for any contrast, confirming that animals of the three experimental groups were very similar pre-infection.

#### Classification of animals based on protein profiles

For classification of animals based on expression of several proteins we used partial least squares with penalized logistic regression [26]. The method combines partial least squares with logistic regression. Partial least squares is both a tool for linear regression and a tool for dimension reduction [27] as we have more explanatory variables, i.e. proteins, than observations. Logistic regression is a common method for binary data using generalized linear models. The method used here combines both and makes it a suitable method for predicting to which categories animals belong based on many predictors [26]. Combining logistic regression with partial least squares has been also applied to disease classification in humans [28]. Here we used the functions rpls (for two-group classification) and mrpls (for three-group classification) from R-package plsgenomics [26]. The parameters lambda and the number of latent variables were determined using cross-validation. The ridge partial least squares method was applied in two ways: 1. by using all proteins and 2. by preselecting the top n proteins with the lowest p-values of ANOVA.

To assess the accuracy of classification we performed a leave-one-out cross-validation, so that every record was left out once from the training set and was predicted based on the others being in the training set. When we used data of different days all records of one animal were used as validation set and the remaining as training set to prevent that the animal itself could have one record as training and another in the validation set. When using preselected proteins, the significance was based on the training set only to prevent the data from the validation animal effecting the preselection of proteins.

Data from day five and day 19 were used both separately and combined for analyses concerning two-group classification (that is distinguishing either infected from non-infected animals, or animals from each disease group (PCV2/PPV, PCV2/PRRSV) versus control animals). For the three-group classification data of day five and 19 were combined. The accuracy of classification was given as number of correctly identified animals. In addition, sensitivity and specificity were calculated for two-way classifications, but not for three-way classifications because sensitivity and specificity are not suitable for that situation. All analyses are summarized in Table 1.

## Authors' contributions

MGJK coordinated the study, participated in its design and harmonized the drafting of the manuscript. HAM performed the statistical analyses and participated in the drafting of the manuscript. NSZ conceived of the animal experiment, provided the serum samples and participated in the drafting of the manuscript. LK performed the SELDI-TOF analyses and participated in the drafting of the manuscript. MAS conceived of the study, participated in its design and participated in the drafting of the manuscript. All authors read and approved the final manuscript.
